# HIV pre‐exposure prophylaxis programme preferences among sexually active HIV‐negative transgender and gender diverse adults in the United States: a conjoint analysis

**DOI:** 10.1002/jia2.26211

**Published:** 2024-02-08

**Authors:** Dovie L. Watson, Louis Listerud, Ryan A. Drab, Willey Y. Lin, Florence Marie Momplaisir, José A. Bauermeister

**Affiliations:** ^1^ Department of Medicine (Infectious Diseases) University of Pennsylvania Perelman School of Medicine Philadelphia Pennsylvania USA; ^2^ Department of Family and Community Health University of Pennsylvania Philadelphia Pennsylvania USA

**Keywords:** choice behaviour, decision‐making, HIV, pre‐exposure prophylaxis, sexual and gender minorities, transgender persons

## Abstract

**Introduction:**

Current implementation efforts have failed to achieve equitable HIV pre‐exposure prophylaxis (PrEP) provision for transgender and gender‐diverse (trans) populations. We conducted a choice‐based conjoint analysis to measure preferences for key attributes of hypothetical PrEP delivery programmes among a diverse online sample predominantly comprised of transmasculine and nonbinary individuals in the United States.

**Methods:**

Between April 2022 and June 2022, a national online survey with an embedded conjoint analysis experiment was conducted among 304 trans individuals aged ≥18 years in the United States to assess five PrEP programme attributes: out‐of‐pocket cost; dispensing venue; frequency of visits for PrEP‐related care; travel time to PrEP provider; and ability to bundle PrEP‐related care with gender‐affirming hormone therapy services. Participants responded to five questions, each of which presented two PrEP programme scenarios and one opt‐out option per question and selected their preferred programme in each question. We used hierarchical Bayes estimation and multinomial logistic regression to measure part‐worth utility scores for the total sample and by respondents’ PrEP status.

**Results:**

The median age was 24 years (range 18–56); 75% were assigned female sex at birth; 54% identified as transmasculine; 32% as nonbinary; 14% as transfeminine. Out‐of‐pocket cost had the highest attribute importance score (44.3%), followed by the ability to bundle with gender‐affirming hormone therapy services (18.7%). Minimal cost‐sharing ($0 out‐of‐pocket cost) most positively influenced the attribute importance of cost (average conjoint part‐worth utility coefficient of 2.5 [95% CI 2.4−2.6]). PrEP‐experienced respondents preferred PrEP delivery in primary care settings (relative utility score 4.7); however, PrEP‐naïve respondents preferred pharmacies (relative utility score 5.1).

**Conclusions:**

Participants preferred programmes that offered PrEP services without cost‐sharing and bundled with gender‐affirming hormone therapy services. Bolstering federal regulations to cover PrEP services and prioritizing programmes to expand low‐barrier PrEP provision are critical to achieving equitable PrEP provision. Community‐engaged implementation research conducted by and in close collaboration with trans community stakeholders and researchers are needed to streamline the design of patient‐centred PrEP programmes and develop implementation strategies that are salient to the diverse sexual health needs of trans patients.

## 1 INTRODUCTION

Social, structural and systemic barriers, including social marginalization, stigma and structural oppression, sustain inequitable HIV pre‐exposure prophylaxis (PrEP) care continuum outcomes in the United States among transgender and gender‐diverse (trans) populations, which include communities of transfeminine, transmasculine, nonbinary and other gender‐diverse individuals whose gender identity or expression differ from cultural expectations [1, 2, 3, 4, 5]. The U.S. Centers for Disease Control and Prevention (CDC) estimated an overall HIV prevalence of 9.2% among trans people in the United States—14% for transgender women and 3% for transgender men [5, 6, 7]. Eligibility criteria outlined in the 2014 and 2017 CDC PrEP guidelines were not specifically inclusive of trans populations [8, 9]. However, if one considers eligibility based on sexual practice indications alone, studies found one‐quarter of transgender men, and more than half of transgender women aged 18−29 years were eligible for PrEP. However, only 10% of PrEP‐eligible transgender men and one‐third of PrEP‐eligible transgender women reported a history of PrEP use [7, 10, 11].

Revised 2021 CDC PrEP guidelines now recommend clinicians inform all sexually active individuals about PrEP and offer PrEP to those at risk of HIV acquisition due to socio‐behavioural factors (e.g. condomless sex with a person of unknown HIV status), structural factors (e.g. member of a community with high HIV prevalence) or expressed interest [12]. In light of these recommendations, tailored implementation strategies to achieve equitable PrEP delivery for trans populations are needed [3, 13]. Regrettably, implementation studies rarely enrol trans participants in numbers sufficient to yield reliable estimates—transfeminine participants are often subsumed into study cohorts comprised predominantly of cisgender sexual minority men, while transmasculine and nonbinary individuals are rarely enrolled at all [13, 14, 15, 16, 17, 18] Limited inclusion of trans participants in implementation research will hinder efforts to develop strategies that are salient and relevant to trans individuals who might otherwise benefit from PrEP due to their current sexual practices or personalized HIV prevention needs and preferences [3, 13, 15, 19]. However, prior research with trans participants across other fields of research may inform this work. Recent quantitative studies have found that anti‐trans stigma, the dearth of trans‐inclusive sexual health programmes and medical mistreatment pose significant barriers to PrEP‐related care [2, 10, 15, 20–24]. Qualitative studies have aligned with these findings and further described how structural factors such as anti‐trans discrimination and disenfranchisement (e.g. poverty, reduced educational opportunities, unstable employment, housing insecurity and carceral system involvement), and limited access to low‐barrier, low‐cost comprehensive healthcare (e.g. gender‐affirming hormone therapy [GAHT], management of chronic medical conditions, sexual health, behavioural health services) impact access to health services and inform PrEP delivery preferences [19, 25–32].

To address these implementation challenges, researchers have integrated behavioural and social sciences research and consumer psychology strategies to inform the design of high‐value PrEP programmes that meet the needs of the intended population [33, 34]. Conjoint analysis (CJA) is an experimental survey technique used to measure respondents’ preferences and the degree of importance respondents assign to specific product attributes when presented with a limited number of profiles (scenarios) comprised of different combinations of attributes [35, 36, 37, 38]. Using CJA, researchers quantitatively measure which combination is associated with product acceptability, relative attribute importance, the most preferred combination and trade‐offs respondents make when selecting their most preferred profile [33, 39–44]. CJA could be leveraged to streamline the design of patient‐centred PrEP programmes for trans patients and ensure trans patients have a meaningful choice of programmes for PrEP‐related care [43, 44, 45, 46, 47]. The objective of this study was to use a choice‐based CJA to measure preferences for the attributes of hypothetical PrEP programmes among a diverse online sample predominantly comprised of transmasculine and nonbinary adults in the United States.

## 2 METHODS

### 2.1 Setting and recruitment

Trans adults in the United States were recruited to complete an online self‐administered survey with an embedded conjoint experiment between April 2022 and June 2022. Recruitment strategies were developed in consultation with our virtual community advisory board (CAB) comprised of six trans adults. Potential participants were recruited via advertisements (64%) on social media (i.e. trans‐specific organizations and groups on Meta, Reddit, etc.), LGBTQ+ and transgender health listservs and word of mouth (11%), and email invitations to trans individuals who previously screened for studies within our research group (25%). Eligibility criteria included: (1) aged ≥18 years; (2) self‐identified as transgender, nonbinary and/or of transgender experience; (3) negative or unknown HIV status; (4) sexual activity in the past 6 months or chlamydia, gonorrhoea or syphilis diagnosis in the past 12 months; (5) U.S. residency; (6) English fluency; and (7) internet access.

Study procedures were approved by the University of Pennsylvania Institutional Review Board. Potential participants completed an online Qualtrics eligibility screener. Research staff reviewed screeners to confirm alignment between respondents’ description of their gender identity (open‐ended free text), sex assigned at birth (exclusive categorical variable) and gender identity (“select all that apply” categorical variable). Unique Qualtrics links were emailed to eligible respondents, and electronic informed consent was obtained from all participants prior to completing the online Qualtrics survey. We used embedded Qualtrics anti‐fraud and duplicate entry metrics, CAPTCHA and other best practices to identify duplicate, falsified and suspicious entries by manually reviewing respondents’ data, including IP addresses, survey completion time, cross‐checking zip code and latitude and longitude coordinates, email addresses and telephone numbers [48, 49, 50]. Participants received a $25 Amazon gift card for survey completion.

### 2.2 Measures

The full survey instrument was developed by the authors, refined based on feedback from the CAB and pretested by CAB members. The parent study assessed the acceptability of next‐generation PrEP modalities and whether participants’ preferences were associated with gender affirmation, sexual behaviours, and barriers and facilitators to healthcare utilization. The survey included an embedded CJA experiment and measures related to socio‐demographic characteristics; social, legal and medical gender affirmation; sexual behaviours; knowledge, beliefs and experiences related to PrEP, HIV/STI prevention and reproductive health. The present research focused on describing the CJA results.

### 2.3 Assigning PrEP programme attributes

Each programme scenario (conjoint) included five attributes selected a priori by the research team based on expert consultation and a literature review of factors shown to influence PrEP delivery among U.S. priority populations [19, 27, 33, 42, 43, 51–59]. Attributes were hypothesized to be independent from each other and relevant for developing programmes to accommodate next‐generation PrEP modalities in development [60, 61, 62]. Attributes included (1) **out‐of‐pocket cost** ($0, $30, $150); (2) **dispensing venue** (pharmacy pick‐up, HIV/STI clinic, primary care or general practitioner medical office); (3) **frequency of visits for PrEP‐related services** (every 2 months, every 3 months, every 6 months); (4) **travel time from PrEP provider** (25 minutes, 60 minutes); and (5) **ability to bundle with GAHT services** (yes, no). (See Table S1.)

### 2.4 CJA procedures using paired comparison methods

**Figure 1:**
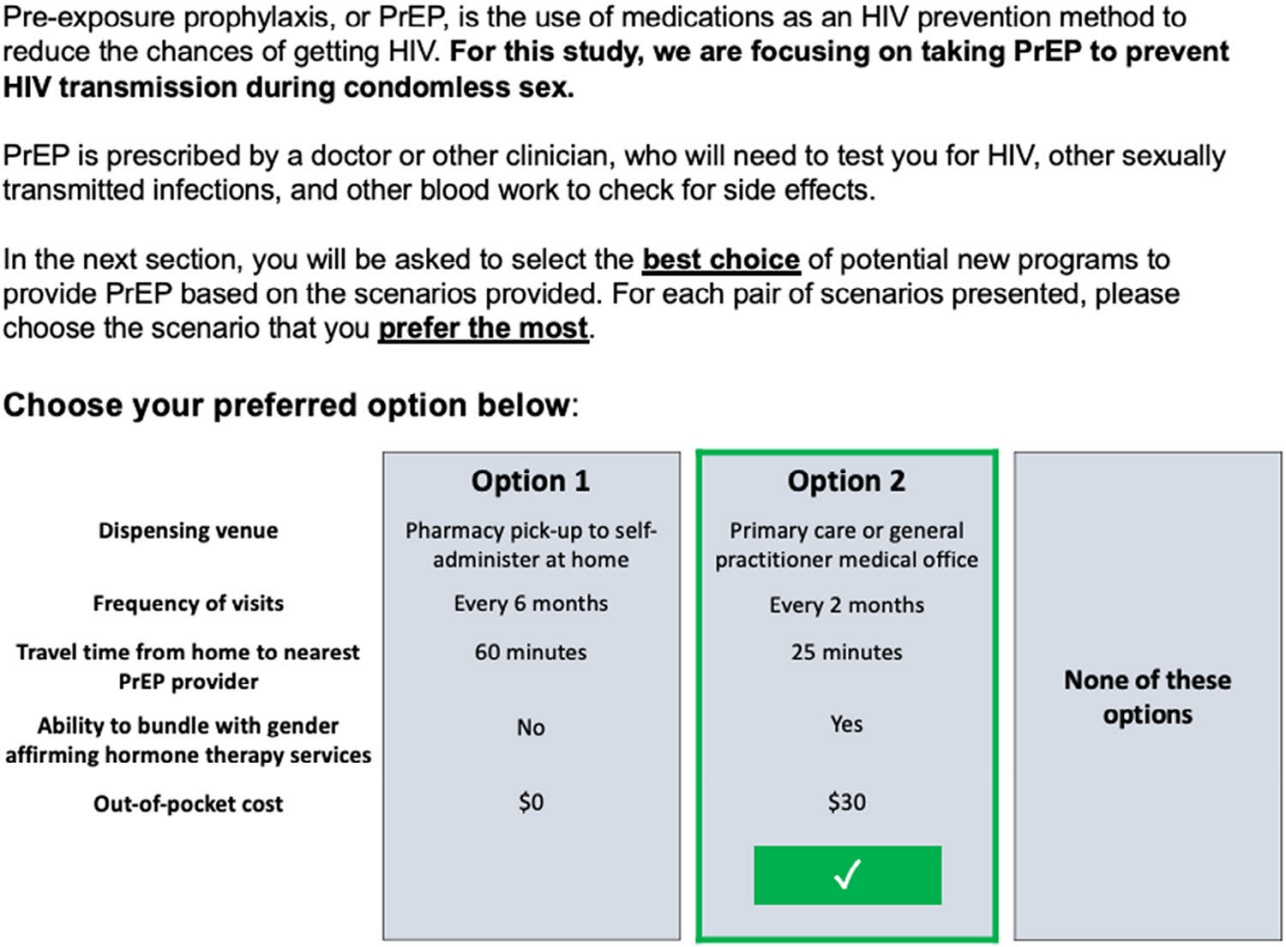
Example of a choice‐based conjoint analysis task. Each task presents two hypothetical HIV pre‐exposure prophylaxis (PrEP) programme profiles (Option 1 and Option 2) and the third option to opt‐out. Each PrEP programme profile presents one level of the five attributes examined in this analysis: dispensing venue, frequency of visits for PrEP‐related care, travel time to PrEP provider, ability to bundle with gender‐affirming hormone therapy services and out‐of‐pocket cost.

The CJA presented a brief description of PrEP followed by five questions, each of which presented two conjoints and one opt‐out option per question, and asked respondents to select their preferred PrEP programme in each question (see Figure 1). Attributes were presented randomly to minimize effect bias. We examined the value participants assigned to different attributes as quantitatively measured by each attribute's **importance score** (i.e. *between* attribute comparison contributing to decision‐making between scenarios), **average utility score** (i.e. *within* attribute comparison of participants’ preferences across levels) and **relative utility score** (i.e. the likelihood that inclusion of this attribute would enhance the optimal “PrEP programme”). The CJA was executed within the proprietary Qualtrics® CJA program (Qualtrics, Provo, UT, 2020), which uses a fractional factorial design with an asymmetric orthogonal main effects plan to reduce the total number of scenarios presented to each participant while presenting each attribute level an approximately equal number of times [35, 37, 63]. In accordance with the rule proposed by Orme for choice‐based conjoint analyses [64], we calculated a sample size of 300 respondents for aggregate‐level estimation of main effects using the following equation:

$$N\geq \frac{\left(multiplier\times c\right)}{\left(t\times a\right)},$$

where N is the minimum number of respondents; *multiplier* = 1000; c=3, largest number of levels for any attribute; t=5, the number of questions; and a=2; the number of choices per question.

### 2.5 Conjoint analyses

The Qualtrics® CJA programme used hierarchical Bayes estimation and multinomial logistic regression to measure each respondent's part‐worth utility scores (regression coefficients) and to characterize attribute importance and programme preferences. Part‐worth utility scores were zero‐centred ordinal scores for each attribute level with a negative score indicating a less preferred level and a positive score indicating a more preferred level [35]. Utility scores were used to calculate attribute metrics for the total study population and by respondents’ PrEP status (PrEP‐naïve vs. PrEP‐experienced). The experimental design (a main‐effects design) did not allow interactions among attributes tested [37].

### 2.6 Descriptive statistics

We computed descriptive statistics for the study population and comparisons across participant characteristics using Fisher's exact tests for categorical variables and Wilcoxon rank sum tests for continuous variables by PrEP status. *p*‐values < 0.05 were considered statistically significant. Statistical analyses not performed in Qualtrics were performed using Stata version 15 (StataCorp LLC, College Station, TX, 2017).

## 3 RESULTS

### 3.1 Sample characteristics

Demographic and other characteristics are shown in Table 1 for 304 respondents, overall and by PrEP status. The median age was 24 years (range 18–56). Overall, 228 (75%) were assigned female sex at birth; 175 (58%) identified as White; 47 (15%) as Hispanic or Latinx/e; and 33 (11%) as Black. More than half (164, 54%) identified as transmasculine, trans men and/or men; 42 (14%) as transfeminine, trans women and/or women; and 99 (32%) as nonbinary, genderqueer or another gender identity that was not transfeminine or transmasculine. One‐fifth of respondents reported prior or current PrEP use, and 209 (87%) PrEP‐naïve respondents reported prior awareness of PrEP. All respondents answered each CJA question, the results of which are shown in Table 2.

**Table 1:** Characteristics of an online sample of HIV‐negative sexually active transgender and gender‐diverse adults in the United States by respondents’ HIV pre‐exposure prophylaxis (PrEP) status (*N* = 304)

Characteristics	Total (*N* = 304)	PrEP‐naïve (*n* = 241)	PrEP‐experienced (*n* = 63)	*p*‐value
Age, median (interquartile range), years	24 (21−29)	23 (20−28)	29 (24−32)	<0.001
Sex assigned at birth				<0.001
Female	228 (75%)	196 (81%)	32 (51%)	
Male	71 (23%)	42 (17%)	29 (46%)	
Prefer not to say	5 (2%)	3 (1%)	2 (3%)	
Gender identity				0.22
Transfeminine, trans woman and/or woman	42 (14%)	32 (13%)	10 (16%)	
Transmasculine, trans man and/or man	164 (54%)	136 (56%)	28 (44%)	
Nonbinary, genderqueer, agender or another gender identity not transfeminine or transmasculine	98 (32%)	73 (30%)	25 (40%)	
Primary sexual identity				0.03
Queer	108 (36%)	87 (36%)	21 (33%)	
Bisexual	74 (24%)	62 (26%)	12 (19%)	
Gay	58 (19%)	37 (15%)	21 (33%)	
Pansexual	28 (9%)	26 (11%)	2 (3%)	
Lesbian	19 (6%)	16 (7%)	3 (5%)	
Another sexual identity	17 (6%)	13 (5%)	4 (6%)	
Race and ethnicity				0.03
Non‐Latinx/e White	175 (58%)	141 (59%)	34 (54%)	
Non‐Latinx/e Black or African American	33 (11%)	19 (8%)	14 (22%)	
Non‐Latinx/e More than one race	29 (10%)	26 (11%)	3 (5%)	
Non‐Latinx/e Another race	20 (7%)	16 (7%)	4 (6%)	
Hispanic, Latinx/e or of Spanish origin (any race)	47 (15%)	39 (16%)	8 (13%)	
Educational attainment				0.62
Not a high school graduate	7 (2%)	5 (2%)	2 (3%)	
High school graduate	166 (55%)	135 (56%)	31 (49%)	
College graduate	101 (33%)	79 (33%)	22 (35%)	
Graduate or professional degree	30 (10%)	22 (9%)	8 (13%)	
Residence by U.S. Census Region				0.27
South	100 (33%)	76 (32%)	24 (38%)	
Northeast	82 (27%)	62 (26%)	20 (32%)	
West	66 (22%)	54 (22%)	12 (19%)	
Midwest	56 (18%)	49 (20%)	7 (11%)	
Employment status				<0.01
Full time ( ≥ 35 hours per week)	144 (47%)	106 (44%)	38 (60%)	
Part time (< 35 hours per week)	70 (23%)	64 (27%)	6 (10%)	
Unemployed	52 (17%)	44 (18%)	8 (13%)	
Student	21 (7%)	16 (7%)	5 (8%)	
Another employment status	17 (6%)	11 (5%)	6 (10%)	
Health insurance coverage status				<0.01
Insurance paid by study participant	154 (51%)	112 (46%)	42 (67%)	
Insurance paid by family member or guardian	124 (41%)	109 (45%)	15 (24%)	
No insurance or unsure of insurance status	26 (9%)	20 (8%)	6 (10%)	
Had a primary care provider	236 (78%)	181 (75%)	55 (87%)	0.04
Reported taking gender‐affirming hormone therapy	152 (50%)	126 (52%)	26 (41%)	0.16
Obtained ≥ 1 gender‐affirming surgical procedure	93 (31%)	74 (31%)	19 (30%)	1.00
Ever diagnosed with a bacterial sexually transmitted infection	48 (16%)	26 (11%)	22 (35%)	<0.001
Ever had sex in exchange for money, food, drugs and so on	60 (20%)	41 (17%)	19 (30%)	0.03

*Note*: Comparisons were made by respondents’ PrEP status using Fisher's exact tests for categorical variables and Wilcoxon rank sum tests for continuous variables. Statistical significance: *p*‐value < 0.05, two‐tailed. Data are presented as median (interquartile range) for continuous measures, and *n* (%) for categorical measures. PrEP‐experienced = report current or prior PrEP use. Gender‐affirming hormone therapy = testosterone, oestradiol, spironolactone, finasteride. Gender‐affirming surgical procedure = genital surgery (e.g. phalloplasty, vaginoplasty); chest surgery (e.g. augmentation, mastectomy); another surgery (e.g. tracheal shaving, facial surgery). Bacterial sexually transmitted infection = *Neisseria gonorrhoeae* (gonorrhoea), *Chlamydia trachomatis* (chlamydia), *Treponema pallidum* (syphilis).

**Table 2:** Results of choice‐based conjoint analysis experiment for online sample of HIV‐negative sexually active transgender and gender‐diverse adults in the United States by respondents’ HIV pre‐exposure prophylaxis (PrEP) status (*N* = 304)

	Total sample (*N* = 304)			PrEP‐naïve participants (*n* = 241)			PrEP‐experienced participants (*n* = 63)		
**Program**me **attribute**	Average utility coefficient (95% CI)	Relative utility score	Attribute importance	Average utility coefficient (95% CI)	Relative utility score	Attribute importance	Average utility coefficient (95% CI)	Relative utility score	Attribute importance
**Out‐of‐pocket cost (USD)**			44.3%			44.9%			43.1%
$0	2.5 (2.37, 2.62)	20.0		2.6 (2.44, 2.70)	20.3		2.2 (1.87, 2.54)	19.2	
$30	0.5 (0.53, 0.56)	4.4		0.5 (0.54, 0.56)	4.3		0.5 (0.50, 0.58)	4.7	
$150	−3.0 (−3.17, −2.91)	−24.3		−3.1 (−3.26, −2.98)	−24.6		−2.7 (−3.10, −2.38)	−23.9	
**Able to bundle with GAHT services**			18.7%			19.2%			16.1%
Yes	1.1 (1.04, 1.21)	9.3		1.2 (1.13, 1.31)	9.6		0.8 (0.56, 0.97)	8.0	
No	−1.1 (−1.21, −1.04)	−9.3		−1.2 (−1.31, −1.13)	−9.6		−0.8 (−0.97, −0.56)	8.0	
**Travel time to PrEP provider**			13.3%			12.8%			14.9%
25 minutes	0.8 (0.81, 0.85)	6.6		0.8 (0.80, 0.84)	6.4		0.9 (0.83, 0.91)	7.5	
60 minutes	−0.8 (−0.85, −0.81)	−6.6		−0.8 (−0.84, −0.80)	−6.4		−0.9 (−0.91, −0.83)	−7.5	
**Dispensing venue**			12.7%			12.3%			13.6%
Pharmacy	0.4 (0.35, 0.53)	5.0		0.5 (0.40, 0.59)	5.1		0.2 (−0.02, 0.45)	4.2	
Primary care office	0.2 (0.19, 0.25)	2.7		0.2 (0.18, 0.24)	2.2		0.3 (0.18, 0.32)	4.7	
HIV/STI clinic	−0.7 (−0.73, −0.59)	−7.7		−0.7 (−0.78, −0.63)	−7.2		−0.5 (−0.65, −0.28)	−8.9	
**Frequency of visits for PrEP‐related care**			11.1%			10.8%			12.3%
Every 2 months	−0.6 (−0.63, −0.53)	−5.0		−0.6 (−0.63, −0.52)	−4.9		−0.6 (−0.71, −0.48)	−5.7	
Every 3 months	−0.2 (−0.17, −0.15)	−1.0		−0.2 (−0.17, −0.16)	−1.0		−0.1 (−0.16, −0.13)	−1.0	
Every 6 months	0.7 (0.69, 0.79)	6.0		0.7 (0.69, 0.79)	5.9		0.7 (0.63, 0.85)	6.6	

*Notes*: Average utility coefficients with 95% confidence intervals (CI) are from multinominal regression models. Part‐worth utility scores were zero‐centred ordinal scores for each attribute level with a negative score indicating a less preferred level and a positive score indicating a more preferred level. Thin lines separate the different attributes assessed.

**Abbreviations**: CI, confidence interval; GAHT, gender‐affirming hormone therapy; STI, sexually transmitted infection; USD, United States Dollar.

#### 3.1.1 Attribute importance scores

**Figure 2:**
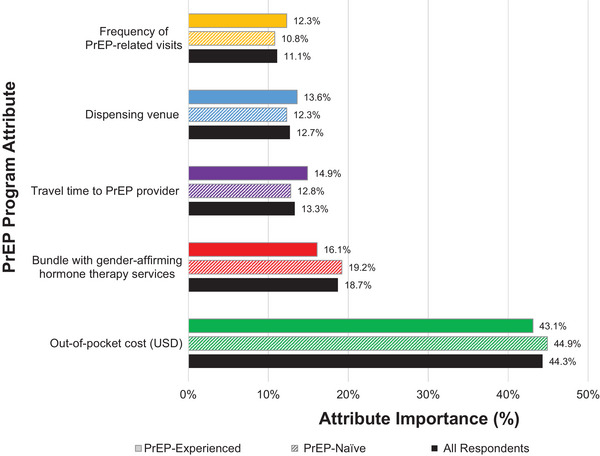
Utility‐based PrEP programme attribute importance scores among all respondents and by respondents’ PrEP status. *Notes*: Attribute importance is the measurement of the distance between the best and worst levels of each attribute and expressed as a percentage. Attribute importance represents the degree to which a given attribute influenced respondents’ preferred programme selection with higher scores indicating greater weight in respondents’ decision‐making process. Abbreviations: PrEP, pre‐exposure prophylaxis; USD, United States Dollar.

Out‐of‐pocket cost had the highest importance score (44.3%), indicating that cost was the most influential attribute during respondents’ decision‐making process when selecting their preferred PrEP programme (see Figure 2). The ability to bundle PrEP‐related care with GAHT services (18.7%) was the second most important attribute, followed by travel time to PrEP provider (13.3%), dispensing venue (12.7%) and frequency of visits for PrEP‐related care (11.1%). The magnitudes and order of attribute importance scores were similar for PrEP‐experienced and PrEP‐naïve respondents. The ability to bundle GAHT services with PrEP‐related care had the largest difference in importance score: 16.1% for PrEP‐experienced respondents compared to 19.2% for PrEP‐naïve respondents.

#### 3.1.2 Average conjoint part‐worth utility scores (coefficients)

**Figure 3:**
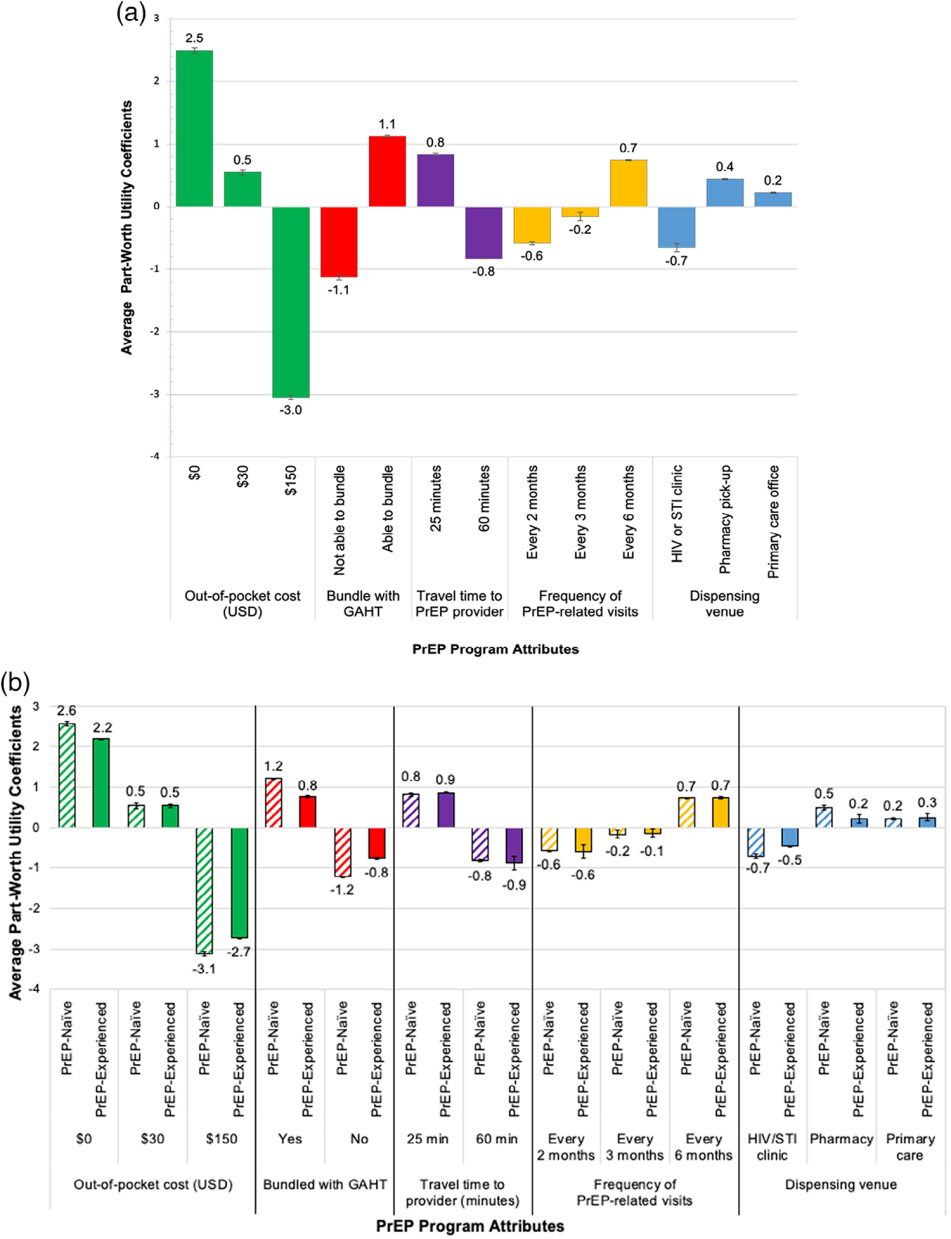
(a) Average part‐worth utility scores (coefficients) of HIV pre‐exposure prophylaxis programme attributes and corresponding levels across all respondents (*N* = 304). (b) Average part‐worth utility scores (coefficients) of HIV pre‐exposure prophylaxis programme attributes and corresponding levels by respondents’ PrEP status (*N* = 304). *Notes*: Average part‐worth utility scores (coefficients) of PrEP programme attributes and corresponding levels with 95% confidence intervals are from multinominal regression models. Average part‐worth utility coefficient represents the degree to which a given level contributed to an attribute's overall attribute importance. Part‐worth utility scores were zero‐centred ordinal scores for each attribute level with a negative score indicating a less preferred level and a positive score indicating a more preferred level. Thin lines separate the different attributes assessed. Abbreviations: GAHT, gender‐affirming hormone therapy; PrEP, pre‐exposure prophylaxis; STI, sexually transmitted infection; USD, United States Dollar.

Average conjoint part‐worth utility coefficients [95% confidence interval] among all respondents and by respondents’ PrEP status are shown in Figures 3a and b, respectively. Across all attributes, $0 out‐of‐pocket cost (2.5 [95% CI 2.4−2.6]) most positively influenced the attribute importance of cost during respondents’ decision‐making process. The ability to bundle with GAHT services (1.1 [95% CI 1.0−1.2]), 25‐minute travel time to the PrEP provider (0.8 [95% CI 0.8−0.8]), attending visits for PrEP‐related services every 6 months (0.7 [95% CI 0.7−0.8]) and PrEP dispensation from the pharmacy (0.4 [95% CI 0.3−0.5]) were the preferred levels for each attribute among all respondents. Higher out‐of‐pocket cost ($150) most negatively influenced the attribute importance of cost (−3.0 [95% CI −3.2 to −2.9]), followed by the inability to bundle with GAHT services (−1.1 [95% CI −1.2 to −1.0]), 60‐minute travel time (−0.8 [95% CI −0.8 to −0.8]), PrEP dispensation from an HIV/STI clinic (−0.7 [95% CI −0.7 to −0.6]) and attending visits every 2 months (−0.6 [95% CI −0.7 to −0.6]).

#### 3.1.3 Relative utility scores for each attribute level

**Figure 4:**
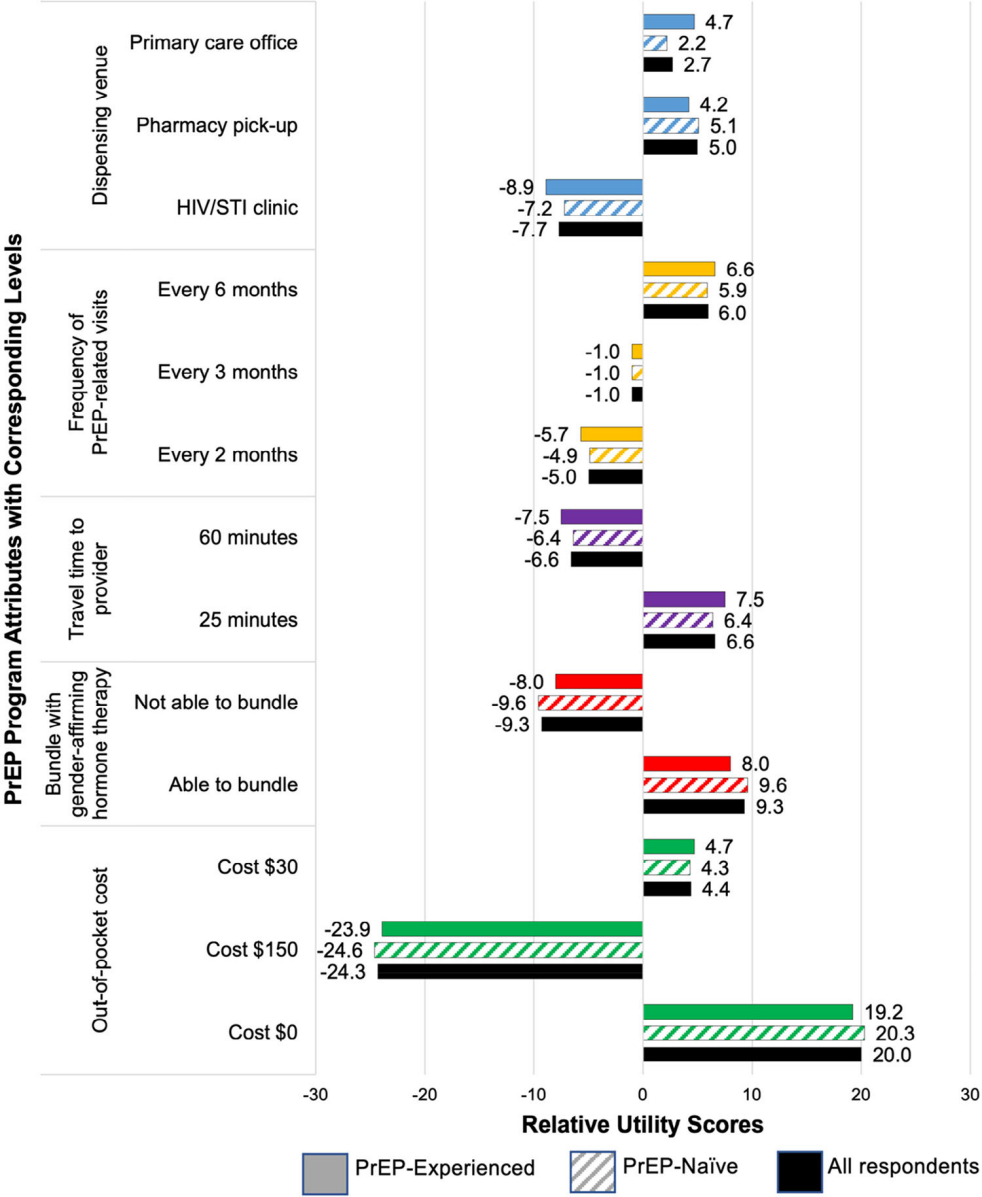
Relative utility scores for each attribute level among all respondents and by respondents’ HIV pre‐exposure prophylaxis status. *Notes*: Within each attribute, the relative utility score of each attribute level represents the degree to which respondents preferred a PrEP programme that had that attribute level. Relative utility scores were zero‐centred ordinal scores for each level with a negative score indicating a less preferred level and a positive score indicating a more preferred level. The higher the positive relative utility score, the more that level enhanced the PrEP programme by being present. Abbreviations: GAHT, gender‐affirming hormone therapy; PrEP, pre‐exposure prophylaxis; STI, sexually transmitted infection; USD, United States Dollar.

As shown in Figure 4, PrEP programmes with $0 out‐of‐pocket cost had the highest relative utility score (20.0) among respondents overall, indicating those programmes were strongly preferred compared to programmes that offered services with $30 out‐of‐pocket cost (4.4) or $150 out‐of‐pocket cost (−24.3). Among respondents overall, relative utility scores were higher for programmes that provided bundled GAHT services, had shorter travel times to providers and required less frequent visits for PrEP‐related services. Relative utility scores for dispensing venue differed by PrEP status. PrEP‐experienced respondents slightly preferred PrEP service delivery in primary care settings (4.7) compared to pharmacies (4.2); however, PrEP‐naïve respondents more strongly preferred pharmacies (5.1) compared to primary care settings (2.2).

## 4 DISCUSSION

This study examined PrEP programme preferences among an online sample predominantly comprised of transmasculine and nonbinary individuals in the United States and quantified the degree to which key attributes contributed to the preferability of PrEP programmes. The findings may inform the design of implementation strategies to enhance the acceptability of PrEP programmes that serve this priority population.

Out‐of‐pocket cost was the most important attribute driving respondents’ PrEP programme preferences across all attributes examined. Our findings are consistent with studies conducted with other priority populations, which found cost‐sharing for PrEP‐related care to be a key structural barrier to PrEP initiation and persistence [23, 43, 55, 57, 65–71]. In accordance with the U.S. Patient Protection and Affordable Care Act (ACA), most health insurance plans are required to cover the cost of PrEP‐related services (i.e. medication, labs and medical visits for PrEP‐specific care) without cost‐sharing. However, the 2022 U.S. federal court ruling in *Braidwood Management v. Becerra* challenged the ACA mandate to provide PrEP coverage without cost‐sharing [72]. If the ruling is upheld and the mandate is overturned, this structural disinvestment in PrEP‐related care will likely disproportionately exacerbate PrEP inequities among trans populations. Bolstering federal regulations that cover PrEP and prioritizing programmes and policies to expand low‐barrier PrEP provision will be critical to achieving PrEP equity [73, 74]. Importantly, socio‐economic deprivation resulting from stigma and discrimination also impedes people's access to the resources needed to support their wellbeing, particularly among trans people experiencing multiple intersecting forms of oppression [75, 76]. For example, anti‐trans stigma has been shown to disrupt people's educational trajectories and adversely affect educational attainment in adulthood, subsequently limiting access to employment opportunities and stable housing, which in turn perpetuates high rates of poverty and financial precarity relative to cisgender people [30, 70, 77–86]. This precarity may also lead to participation in sex work—thus conveying increased vulnerability to HIV transmission if engaging in condomless sex with clients and increased risk of surveillance, profiling, carceral system involvement due to the widespread criminalization of sex work [84, 87]. Future implementation strategies in the United States should prioritize PrEP provision with limited out‐of‐pocket cost—ideally no out‐of‐pocket cost—to PrEP users, including those who are uninsured.

Half of the respondents reported they had not used GAHT as a component of their gender affirmation. Our findings are consistent with prior work that found not all trans adults seek gender affirmation services within formal medical institutions due to a multitude of reasons, including experiences of anti‐trans discrimination, administrative burden, legislative state‐level restrictions on trans healthcare, refusal of care from medical providers, preference for do‐it‐yourself hormone management or simply lack of desire to do so [21, 88–93]. Importantly, the ability to bundle PrEP‐related services with GAHT services was still highly preferred and the second most important attribute assessed in this study. This aligns with prior studies, which found significant associations between the receipt of gender‐affirming medical services and the use of sexual health services including PrEP [2, 19, 94]. Incorporation of GAHT services into PrEP‐related care will require broader integration of transgender‐inclusive sexual health services and training in gender‐affirming healthcare delivery among healthcare providers, including trainees [26, 95–97]. Future implementation efforts should also consider how best to integrate GAHT services to ensure PrEP‐related care is salient to trans patients’ comprehensive sexual health needs.

HIV/STI clinics were the least preferred dispensing venue among both PrEP‐naïve and PrEP‐experienced respondents. Our findings align with a recent discrete choice experiment with Black cisgender women in the United States, which also found HIV/STI clinics were the least preferred location to obtain PrEP‐related care [98]. In addition, prior studies have found suboptimal rates of PrEP initiation and persistence among STI clinic patients [71, 99–101]. Taken together, these findings suggest that HIV/STI clinics may not be the preferred venue for PrEP delivery for certain subgroups among priority populations. Privacy concerns and stigma associated with accessing services from a clinic recognized by community members as a location that focuses on HIV/STI‐related care may influence this preference. We also found differences in the most preferred dispensing venue by PrEP status. Among PrEP‐naïve respondents, PrEP dispensation from a pharmacy was the most preferable location. In contrast, receipt of PrEP from a primary care office was the most preferable location among PrEP‐experienced respondents. Our findings align with calls from community stakeholders and HIV prevention researchers to integrate PrEP delivery into settings that are not exclusively focused on HIV/STI care [23, 52, 102–111]. Furthermore, the revised 2021 CDC PrEP Guidelines recommend clinicians routinize PrEP discussions with all sexually active patients and incorporate PrEP into other preventive services to ensure greater reach and more equitable access [12]. Further investigation, including qualitative research, is needed to explore the factors driving differential preferences in PrEP provision locations.

Shorter travel time to one's PrEP provider was the third most important attribute among respondents regardless of PrEP status. Lack of geographically accessible medical services is an important barrier to attending medical visits, including visits for PrEP‐related care, among people who reside in rural areas or impoverished neighbourhoods [22, 103, 112–114]. Prior studies also found that even in large metropolitan areas, trans patients often reported that ride‐share services were cost‐prohibitive and travelling by public transportation was time‐consuming or exposed them to discrimination or violence [27, 115, 116]. Increasing geographic accessibility of PrEP‐related services will be a critical systemic facilitator for ensuring equitable PrEP delivery for trans people, particularly those who must navigate additional systemic barriers, such as living in poverty, in rural areas or lack of affordable and safe transportation. Implementation efforts are ongoing to expand PrEP delivery by increasing the number of PrEP providers and diversifying PrEP delivery models (e.g. telemedicine services, pharmacy‐based services and delivery in primary care practices) [3, 27, 52, 73, 74, 80, 109, 110].

Finally, the ability to decrease the frequency of PrEP‐related visits to every 6 months was strongly preferred among all respondents regardless of PrEP status. Several next‐generation PrEP modalities under development may be amenable to this preference [13, 60, 62, 80]. Future studies should be more inclusive and enrol more substantial numbers of trans participants to obtain more reliable findings related to product efficacy, acceptability and implementation opportunities among this priority population. The focus should be directed towards individuals for whom daily oral tenofovir‐based PrEP and bimonthly injectable cabotegravir are not acceptable or feasible.

Some limitations of our study should be noted. First, although the full survey instrument was piloted and revised based on CAB feedback, we were unable to confirm the extent to which a given respondent understood each attribute while selecting their preferred scenarios. Second, participants may not represent those most vulnerable to HIV acquisition. However, our eligibility criteria aligned with the 2021 CDC PrEP clinical guidelines [12]. Third, most participants were recruited via social media and reported high rates of PrEP awareness, which may have introduced sampling bias and limited the generalizability of our findings. Fourth, it is possible that cisgender individuals were misclassified as trans and deemed eligible to participate in this study, thereby introducing bias. Fifth, our CJA examined preferred programme‐level attributes rather than attributes of PrEP products (e.g. adverse drug effects, route of administration, duration of protection). Lastly, respondents’ reported preferences for hypothetical programme attributes may not accurately represent their actual choice behaviours. Future research is needed to ensure implementation efforts are responsive to the evolving landscape of next‐generation PrEP development and service delivery models.

## 5 CONCLUSIONS

PrEP is one of the most revolutionary and highly effective methods of HIV prevention. Due to numerous social, structural and systemic barriers, current delivery models have failed to eliminate the entrenched population‐level PrEP inequities among trans populations in the United States [2]. The PrEP‐related needs and preferences of trans patients—and transmasculine and nonbinary patients specifically—have not been well explored. This study examined PrEP programme preferences among a diverse national online cohort of sexually active trans adults, the majority of whom identified as transmasculine or nonbinary. Key findings included high rates of PrEP awareness, an overwhelming preference for PrEP services without cost‐sharing, a strong preference for PrEP services bundled with GAHT services and differential preferences in PrEP provision locations between PrEP‐naïve and PrEP‐experienced respondents. Community‐engaged implementation and qualitative research conducted by and in collaboration with trans community stakeholders and researchers are needed to obtain a nuanced understanding of the PrEP‐related needs and preferences among this diverse priority population [3]. Future implementation efforts should consider how models of PrEP delivery for next‐generation PrEP may impact scale‐up among current and potential trans‐PrEP users and prioritize strategies that address the complex factors impeding equitable PrEP delivery, which is a public health priority.

## COMPETING INTERESTS

The authors declare that they have no competing interests.

## AUTHORS’ CONTRIBUTIONS

DLW, FMM and JAB had overall responsibility for implementing the study, conceived and designed the study, and developed the study protocol. DLW led the original drafting of the manuscript, contributed to participant recruitment, conducted data collection and data analysis, contributed to funding acquisition and supervised the overall study. LL gave feedback/revisions on earlier versions of the manuscript and contributed to participant recruitment, study implementation and data collection. RAD and WL gave feedback/revisions on earlier versions of the manuscript and contributed to participant recruitment and study implementation. FMM gave feedback/revisions on earlier versions of the manuscript. JAB gave feedback/revisions on earlier versions of the manuscript, conducted data analysis, contributed to funding acquisition, contributed staff resources and research infrastructure, and supervised the overall study.

## FUNDING

This research was supported by a grant from the Penn Center for AIDS Research, which is funded by the National Institute of Allergy and Infectious Diseases under award number P30‐AI‐045008.

## DISCLAIMER

The content of this article is solely the responsibility of the authors and does not necessarily represent the official views of the National Institute of Allergy and Infectious Diseases or the National Institutes of Health.

## INFORMED CONSENT

Inform consent was obtained from all individuals included in the study.

## Supporting information

Supporting Information

## Data Availability

Data are presented in aggregate form within the manuscript. Individual‐level data contain potentially sensitive information and are not suitable for public repository due to ethical restrictions and to protect confidentiality. Reasonable requests to access the final de‐identified data set should be submitted to the corresponding author's email, who will evaluate and approve the request.

## References

[1] Malone J , Reisner SL , Cooney EE , Poteat T , Cannon CM , Schneider JS , et al. Perceived HIV acquisition risk and low uptake of PrEP among a cohort of transgender women with PrEP indication in the eastern and Southern United States. J Acquir Immune Defic Syndr. 2021;88(1):10–18. 34397742 10.1097/QAI.0000000000002726 PMC8371736

[2] Dang M , Scheim AI , Teti M , Quinn KG , Zarwell M , Petroll AE , et al. Barriers and facilitators to HIV pre‐exposure prophylaxis uptake, adherence, and persistence among transgender populations in the United States: a systematic review. AIDS Patient Care STDs. 2022;36(6):236–248. 35687813 10.1089/apc.2021.0236 PMC9242706

[3] Restar A , Minalga BJ , Quilantang MI , Adamson T , Dusic E , van der Merwe LA , et al. Mapping community‐engaged implementation strategies with transgender scientists, stakeholders, and trans‐led community organizations. Curr HIV/AIDS Rep. 2023;20(3):160‐169. 37012537 10.1007/s11904-023-00656-y PMC10071255

[4] Scheim AI , Baker KE , Restar AJ , Sell RL . Health and health care among transgender adults in the United States. Annu Rev Public Health. 2022;43:503–523. 34882432 10.1146/annurev-publhealth-052620-100313

[5] Becasen JS , Denard CL , Mullins MM , Higa DH , Sipe TA . Estimating the prevalence of HIV and sexual behaviors among the US transgender population: a systematic review and meta‐analysis, 2006–2017. Am J Public Health. 2019;109(1):e1–e8. 10.2105/AJPH.2018.304727 PMC6301428 30496000

[6] Centers for Disease Control and Prevention . HIV Surveillance Report, 2019. Atlanta, GA: U.S. Department of Health and Human Services; 2021.

[7] Centers for Disease Control and Prevention . HIV infection, risk, prevention, and testing behaviors among transgender women—national HIV behavioral surveillance, 7 U.S. cities, 2019–2020. Atlanta, GA: U.S. Department of Health and Human Services; 2021.

[8] Centers for Disease Control and Prevention . Preexposure prophylaxis for the prevention of HIV infection in the United States—2017 update: a clinical practice guideline. Atlanta, GA: U.S. Department of Health and Human Services; 2018.

[9] Centers for Disease Control and Prevention . Preexposure prophylaxis for the prevention of HIV infection in the United States—2014—a clinical practice guideline. Atlanta, GA: U.S. Department of Health and Human Services; 2014.

[10] Golub SA , Fikslin RA , Starbuck L , Klein A . High rates of PrEP eligibility but low rates of PrEP access among a national sample of transmasculine individuals. J Acquir Immune Defic Syndr. 2019;82(1):e1–e7. 31232834 10.1097/QAI.0000000000002116 PMC6692190

[11] Kuhns LM , Reisner SL , Mimiaga MJ , Gayles T , Shelendich M , Garofalo R . Correlates of PrEP indication in a multi‐site cohort of young HIV‐uninfected transgender women. AIDS Behav. 2016;20(7):1470–1477. 26336946 10.1007/s10461-015-1182-z PMC4777686

[12] Centers for Disease Control and Prevention . Preexposure prophylaxis for the prevention of HIV infection in the United States–2021 update clinical practice guideline. U.S. Department of Health and Human Services; 2021.

[13] Zamantakis A , Li DH , Benbow N , Smith JD , Mustanski B . Determinants of pre‐exposure prophylaxis (PrEP) implementation in transgender populations: a qualitative scoping review. AIDS Behav. 2023;27(5):1600‐1618. 36520334 10.1007/s10461-022-03943-8 PMC9753072

[14] Del Río‐González AM , Lameiras‐Fernández M , Modrakovic D , Aguayo‐Romero R , Glickman C , Bowleg L , et al. Global scoping review of HIV prevention research with transgender people: transcending from trans‐subsumed to trans‐centred research. J Int AIDS Soc. 2021;24(9):e25786. 34473421 10.1002/jia2.25786 PMC8412127

[15] Teng F , Sha Y , Fletcher LM , Welsch M , Burns P , Tang W . Barriers to uptake of PrEP across the continuum among transgender women: a global scoping review. Int J STD AIDS. 2023;34(5):299–314. 36793197 10.1177/09564624231152781

[16] U.S. Food and Drug Administration . FDA briefing document: meeting of the Antimicrobial Drugs Advisory Committee. Silver Spring, MD: U.S. Department of Health and Human Services; 2019.

[17] Mayer KH , Molina JM , Thompson MA , Anderson PL , Mounzer KC , De Wet JJ , et al. Emtricitabine and tenofovir alafenamide vs emtricitabine and tenofovir disoproxil fumarate for HIV pre‐exposure prophylaxis (DISCOVER): primary results from a randomised, double‐blind, multicentre, active‐controlled, phase 3, non‐inferiority trial. Lancet. 2020;396(10246):239–254. 32711800 10.1016/S0140-6736(20)31065-5 PMC9665936

[18] Asquith A , Sava L , Harris AB , Radix AE , Pardee DJ , Reisner SL . Patient‐centered practices for engaging transgender and gender diverse patients in clinical research studies. BMC Med Res Methodol. 2021;21(1):202. 34598674 10.1186/s12874-021-01328-4 PMC8487157

[19] Klein A , Golub SA . Increasing access to pre‐exposure prophylaxis among transgender women and transfeminine nonbinary individuals. AIDS Patient Care STDs. 2019;33(6):262–269. 31166785 10.1089/apc.2019.0049

[20] Hughto JMW , Pachankis JE , Reisner SL . Healthcare mistreatment and avoidance in trans masculine adults: the mediating role of rejection sensitivity. Psychol Sex Orientat Gend Divers. 2018;5(4):471–481. 30637266 10.1037/sgd0000296 PMC6328255

[21] Jaffee KD , Shires DA , Stroumsa D . Discrimination and delayed health care among transgender women and men: implications for improving medical education and health care delivery. Med Care. 2016;54(11):1010–1016. 27314263 10.1097/MLR.0000000000000583

[22] Newsom KD , Riddle MJ , Carter GA , Hille JJ . They “don't know how to deal with people like me”: assessing health care experiences of gender minorities in Indiana. Transgend Health. 2022;7(5):453–460. 36644487 10.1089/trgh.2021.0027 PMC9829144

[23] Reisner SL , Moore CS , Asquith A , Pardee DJ , Mayer KH . The pre‐exposure prophylaxis cascade in at‐risk transgender men who have sex with men in the United States. LGBT Health. 2021;8(2):116–124. 33567245 10.1089/lgbt.2020.0232 PMC8195872

[24] Kuhns LM , Perloff J , Johnson AK , Paul JL , Pleasant K , Evans K , et al. A cross‐sectional analysis of psychosocial and structural barriers and facilitators associated with PrEP use among a sample of transgender women in Chicago, IL. AIDS Res Ther. 2023;20(1):24. 37085860 10.1186/s12981-023-00516-0 PMC10122350

[25] Nieto O , Fehrenbacher AE , Cabral A , Landrian A , Brooks RA . Barriers and motivators to pre‐exposure prophylaxis uptake among Black and Latina transgender women in Los Angeles: perspectives of current PrEP users. AIDS Care. 2021;33(2):244–252. 32449399 10.1080/09540121.2020.1769835 PMC7680715

[26] Matsuzaka S , Romanelli M , Hudson KD . “Render a service worthy of me”: a qualitative study of factors influencing access to LGBTQ‐specific health services. SSM—Qual Res Health. 2021;1:100019.

[27] Rael CT , Lopez‐Ríos J , McKenna SA , Das D , Dolezal C , Abascal E , et al. Transgender women's barriers, facilitators, and preferences on tailored injection delivery strategies to administer long‐acting injectable cabotegravir (CAB‐LA) for HIV pre‐exposure prophylaxis (PrEP). AIDS Behav. 2021;25(12):4180–4192. 34216284 10.1007/s10461-021-03357-y PMC8254438

[28] Kerman J , Brewer R , Hotton A , Flores R , Devlin SA , Friedman EE , et al. Multi‐level and intersectional stigma experienced by Black transgender women in Chicago: a qualitative study to inform sociostructural interventions for reducing stigma and improving health outcomes. J Racial Ethn Health Disparities. 2023. 10.1007/s40615-023-01853-6 PMC11089070 37957538

[29] Ogunbajo A , Storholm ED , Ober AJ , Bogart LM , Reback CJ , Flynn R , et al. Multilevel barriers to HIV PrEP uptake and adherence among Black and Hispanic/Latinx transgender women in Southern California. AIDS Behav. 2021;25(7):2301–22315. 33515132 10.1007/s10461-021-03159-2 PMC7845787

[30] Glick JL , Lopez A , Pollock M , Theall KP . Housing insecurity and intersecting social determinants of health among transgender people in the USA: a targeted ethnography. Int J Transgend Health. 2020;21(3):337–349. 34993513 10.1080/26895269.2020.1780661 PMC8726680

[31] Horvath KJ , Todd K , Arayasirikul S , Cotta NW , Stephenson R . Underutilization of pre‐exposure prophylaxis services among transgender and nonbinary youth: findings from Project Moxie and TechStep. Transgend Health. 2019;4(1):217–221. 31592151 10.1089/trgh.2019.0027 PMC6778317

[32] Wood S , Gross R , Shea JA , Bauermeister JA , Franklin J , Petsis D , et al. Barriers and facilitators of PrEP adherence for young men and transgender women of color. AIDS Behav. 2019;23(10):2719–2729. 30993479 10.1007/s10461-019-02502-y PMC6790163

[33] Bauermeister JA , Downs JS , Krakower DS . PrEP product acceptability and dual process decision‐making among men who have sex with men. Curr HIV/AIDS Rep. 2020;17(3):161–170. 32297220 10.1007/s11904-020-00497-z PMC7260091

[34] Morton T , Chege W , Swann E , Senn TE , Cleland N , Renzullo PO , et al. Advancing long‐acting and extended delivery HIV prevention and treatment regimens through behavioural science: NIH workshop directions. AIDS. 2021;35(8):1313–1317. 33710013 10.1097/QAD.0000000000002863

[35] Qualtrics XM . Conjoint analysis white paper. 2022. Accessed 1 May 2023. Available at: https://www.qualtrics.com/support/conjoint‐project/getting‐started‐conjoints/getting‐started‐choice‐based/conjoint‐analysis‐white‐paper/

[36] Bridges JF , Hauber AB , Marshall D , Lloyd A , Prosser LA , Regier DA , et al. Conjoint analysis applications in health–a checklist: a report of the ISPOR Good Research Practices for Conjoint Analysis Task Force. Value Health. 2011;14(4):403–413. 21669364 10.1016/j.jval.2010.11.013

[37] Rao VR . Applied conjoint analysis. 1st ed. Heidelberg: Springer Berlin; 2014. p. 389.

[38] Reed Johnson F , Lancsar E , Marshall D , Kilambi V , Mühlbacher A , Regier DA , et al. Constructing experimental designs for discrete‐choice experiments: report of the ISPOR Conjoint Analysis Experimental Design Good Research Practices Task Force. Value Health. 2013;16(1):3–13. 23337210 10.1016/j.jval.2012.08.2223

[39] Phillips KA , Maddala T , Johnson FR . Measuring preferences for health care interventions using conjoint analysis: an application to HIV testing. Health Serv Res. 2002;37(6):1681–1705. 12546292 10.1111/1475-6773.01115 PMC1464051

[40] Browne EN , Montgomery ET , Mansfield C , Boeri M , Mange B , Beksinska M , et al. Efficacy is not everything: eliciting women's preferences for a vaginal HIV prevention product using a discrete‐choice experiment. AIDS Behav. 2020;24(5):1443–1451. 31696371 10.1007/s10461-019-02715-1 PMC6990865

[41] Montgomery ET , Beksinska M , Mgodi N , Schwartz J , Weinrib R , Browne EN , et al. End‐user preference for and choice of four vaginally delivered HIV prevention methods among young women in South Africa and Zimbabwe: the Quatro Clinical Crossover Study. J Int AIDS Soc. 2019;22(5):e25283. 31069957 10.1002/jia2.25283 PMC6506690

[42] Simoni JM , Tapia K , Lee SJ , Graham SM , Beima‐Sofie K , Mohamed ZH , et al. A conjoint analysis of the acceptability of targeted long‐acting injectable antiretroviral therapy among persons living with HIV in the U.S. AIDS Behav. 2020;24(4):1226–1236. 31655915 10.1007/s10461-019-02701-7 PMC7085450

[43] Shrestha R , Karki P , Altice FL , Dubov O , Fraenkel L , Huedo‐Medina T , et al. Measuring acceptability and preferences for implementation of pre‐exposure prophylaxis (PrEP) using conjoint analysis: an application to primary HIV prevention among high risk drug users. AIDS Behav. 2018;22(4):1228–1238. 28695388 10.1007/s10461-017-1851-1 PMC5762432

[44] Beckham SW , Crossnohere NL , Gross M , Bridges JFP . Eliciting preferences for HIV prevention technologies: a systematic review. Patient. 2021;14(2):151–174. 33319339 10.1007/s40271-020-00486-9 PMC7884379

[45] Eshun‐Wilson I , Kim HY , Schwartz S , Conte M , Glidden DV , Geng EH . Exploring relative preferences for HIV service features using discrete choice experiments: a synthetic review. Curr HIV/AIDS Rep. 2020;17(5):467–477. 32860150 10.1007/s11904-020-00520-3 PMC7497362

[46] Gutierrez JI , Dubov A , Altice FL , Vlahov D . Preferences for pre‐exposure prophylaxis among U.S. military men who have sex with men: results of an adaptive choice based conjoint analysis study. Mil Med Res. 2021;8(1):32. 34006328 10.1186/s40779-021-00323-6 PMC8132436

[47] Schieffer RJ , Bryndza Tfaily E , D'Aquila R , Greene GJ , Carballo‐Diéguez A , Giguere R , et al. Conjoint analysis of user acceptability of sustained long‐acting pre‐exposure prophylaxis for HIV. AIDS Res Hum Retroviruses. 2022;38(4):336‐345. 34779227 10.1089/aid.2021.0075 PMC9048179

[48] Bauermeister J , Pingel E , Zimmerman M , Couper M , Carballo‐Diéguez A , Strecher VJ . Data quality in web‐based HIV/AIDS research: handling invalid and suspicious data. Field Methods. 2012;24(3):272–291. 23180978 10.1177/1525822X12443097 PMC3505140

[49] Bauermeister JA . Sexual partner typologies among single young men who have sex with men. AIDS Behav. 2015;19(6):1116–1128. 25358726 10.1007/s10461-014-0932-7 PMC4417101

[50] Lawlor J , Thomas C , Guhin AT , Kenyon K , Lerner MD , Drahota A . Suspicious and fraudulent online survey participation: introducing the REAL framework. Methodol Innov. 2021;14(3):1‐10.

[51] Silapaswan A , Krakower D , Mayer KH . Pre‐exposure prophylaxis: a narrative review of provider behavior and interventions to increase PrEP implementation in primary care. J Gen Intern Med. 2017;32(2):192–198. 27761767 10.1007/s11606-016-3899-4 PMC5264683

[52] Mayer KH , Chan PA , Patel RR , Flash CA , Krakower DS . Evolving models and ongoing challenges for HIV preexposure prophylaxis implementation in the United States. J Acquir Immune Defic Syndr. 2018;77(2):119–127. 29084044 10.1097/QAI.0000000000001579 PMC5762416

[53] Siegler AJ , Bratcher A , Weiss KM . Geographic access to preexposure prophylaxis clinics among men who have sex with men in the United States. Am J Public Health. 2019;109(9):1216–1223. 31318587 10.2105/AJPH.2019.305172 PMC6687234

[54] Sullivan PS , Mena L , Elopre L , Siegler AJ . Implementation strategies to increase PrEP uptake in the South. Curr HIV/AIDS Rep. 2019;16(4):259–269. 31177363 10.1007/s11904-019-00447-4 PMC7117066

[55] Golub SA , Myers JE . Next‐wave HIV pre‐exposure prophylaxis implementation for gay and bisexual men. AIDS Patient Care STDs. 2019;33(6):253–261. 31094576 10.1089/apc.2018.0290 PMC6588121

[56] Owens C , Hubach RD , Williams D , Lester J , Reece M , Dodge B . Exploring the pre‐exposure prophylaxis (PrEP) health care experiences among men who have sex with men (MSM) who live in rural areas of the Midwest. AIDS Educ Prev. 2020;32(1):51–66. 32073310 10.1521/aeap.2020.32.1.51

[57] Kay ES , Pinto RM . Is insurance a barrier to HIV preexposure prophylaxis? Clarifying the issue. Am J Public Health. 2020;110(1):61–64. 31725314 10.2105/AJPH.2019.305389 PMC6893325

[58] Adeagbo O , Harrison S , Qiao S , Li X . Pre‐exposure prophylaxis (PrEP) uptake among Black men who have sex with men (BMSM) in the southern U.S. Int J Environ Res Public Health. 2021;18(18):9715. 34574652 10.3390/ijerph18189715 PMC8470377

[59] Bonacci RA , Van Handel M , Huggins R , Inusah S , Smith DK . Estimated uncovered costs for HIV preexposure prophylaxis in the US, 2018. Health Aff (Millwood). 2023;42(4):546–555. 37011310 10.1377/hlthaff.2022.00523 PMC10206677

[60] Beymer MR , Holloway IW , Pulsipher C , Landovitz RJ . Current and future PrEP medications and modalities: on‐demand, injectables, and topicals. Curr HIV/AIDS Rep. 2019;16(4):349–358. 31222499 10.1007/s11904-019-00450-9 PMC6719717

[61] Markowitz M , Grobler JA . Islatravir for the treatment and prevention of infection with the human immunodeficiency virus type 1. Curr Opin HIV AIDS. 2020;15(1):27–32. 31658118 10.1097/COH.0000000000000599

[62] Flexner C , Owen A , Siccardi M , Swindells S . Long‐acting drugs and formulations for the treatment and prevention of HIV infection. Int J Antimicrob Agents. 2021;57(1):106220. 33166693 10.1016/j.ijantimicag.2020.106220 PMC7790856

[63] Plackett RL , Burman JP . The design of optimum multifactorial experiments. Biometrika. 1946;33(4):305–325.

[64] Orme BK . Getting started with conjoint analysis: strategies for product design and pricing research. 4th ed. Manhattan Beach, CA: Research Publishers LLC; 2020.

[65] Marcus JL , Hurley LB , Dentoni‐Lasofsky D , Ellis CG , Silverberg MJ , Slome S , et al. Barriers to preexposure prophylaxis use among individuals with recently acquired HIV infection in Northern California. AIDS Care. 2019;31(5):536–544. 30304942 10.1080/09540121.2018.1533238 PMC6408235

[66] Shorrock F , Alvarenga A , Hailey‐Fair K , Vickroy W , Cos T , Kwait J , et al. Dismantling barriers and transforming the future of pre‐exposure prophylaxis uptake in young Black and Latinx sexual minority men and transgender women. AIDS Patient Care STDs. 2022;36(5):194–203. 35507322 10.1089/apc.2021.0222 PMC9125574

[67] Rowe K , Theodore DA , Zucker J , Cohensedgh O , LaSota E , Carnevale C , et al. Lost2PrEP: understanding reasons for pre‐exposure prophylaxis and sexual health care disengagement among men who have sex with men attending a sexual health clinic at a large urban academic medical center in New York City. AIDS Patient Care STDs. 2022;36(4):153–158. 35438522 10.1089/apc.2022.0004 PMC9057871

[68] Corneli A , Perry B , Wilson J , Reif S , Gulden C , Hanlen‐Rosado E , et al. Identification of determinants and implementation strategies to increase PrEP uptake among Black same gender‐loving men in Mecklenburg County, North Carolina: the PrEP‐MECK study. J Acquir Immune Defic Syndr. 2022;90(S1):S149–S160. 35703767 10.1097/QAI.0000000000002975 PMC9220775

[69] Bradley E , Forsberg K , Betts JE , DeLuca JB , Kamitani E , Porter SE , et al. Factors affecting pre‐exposure prophylaxis implementation for women in the United States: a systematic review. J Womens Health (Larchmt). 2019;28(9):1272–1285. 31180253 10.1089/jwh.2018.7353

[70] Bonacci RA , Smith DK , Ojikutu BO . Toward greater pre‐exposure prophylaxis equity: increasing provision and uptake for Black and Hispanic/Latino individuals in the U.S. Am J Prev Med. 2021;61(5 Suppl 1):S60–S72. 34686293 10.1016/j.amepre.2021.05.027 PMC8668046

[71] Hojilla JC , Vlahov D , Crouch PC , Dawson‐Rose C , Freeborn K , Carrico A . HIV pre‐exposure prophylaxis (PrEP) uptake and retention among men who have sex with men in a community‐based sexual health clinic. AIDS Behav. 2018;22(4):1096–1099. 29243109 10.1007/s10461-017-2009-x PMC5879003

[72] *Braidwood Management Inc. v. XavierBecerra* (7 September 2022), Civil Action No. 4:20‐cv‐00283‐O.

[73] U.S. Health Resources and Services Administration . Integrating HIV care, treatment & prevention services into primary care—a toolkit for health centers. Rockville, MD: U.S. Department of Health and Human Services; 2017.

[74] Zablotska IB , O'Connor CC . Preexposure prophylaxis of HIV infection: the role of clinical practices in ending the HIV epidemic. Curr HIV/AIDS Rep. 2017;14(6):201–210. 29071519 10.1007/s11904-017-0367-7

[75] Hatzenbuehler ML , Phelan JC , Link BG . Stigma as a fundamental cause of population health inequalities. Am J Public Health. 2013;103(5):813–821. 23488505 10.2105/AJPH.2012.301069 PMC3682466

[76] Wiginton JM , Maksut JL , Scheim AI , Zlotorzynska M , Sanchez TH , Baral SD . Intersecting sexual behavior and gender identity stigmas among transgender women in the United States: burden and associations with sexual health. AIDS Behav. 2023;27(9):3064–3079. 36952112 10.1007/s10461-023-04028-w PMC10034890

[77] Conron KJ , O'Neill KK , Vasquez LA . Educational experiences of transgender people. UCLA School of Law Williams Institute; 2022.

[78] Wilkinson L , Pearson J , Liu H . Educational attainment of transgender adults: does the timing of transgender identity milestones matter? Soc Sci Res. 2018;74:146–160. 29961481 10.1016/j.ssresearch.2018.04.006 PMC6234844

[79] McNamara M , Harsono D , Edelman EJ , Norwood A , Hill SV , Paltiel AD , et al. Braidwood misreads the science: the PrEP mandate promotes public health for the entire community. 2023. Accessed 1 August 2023. Available at: https://law.yale.edu/sites/default/files/documents/pdf/prep_report_final_feb_13_2023_rev.pdf

[80] Philbin MM , Perez‐Brumer A . Promise, perils and cautious optimism: the next frontier in long‐acting modalities for the treatment and prevention of HIV. Curr Opin HIV AIDS. 2022;17(2):72–88. 35225248 10.1097/COH.0000000000000723 PMC8915989

[81] Murry VM , Bradley C , Cruden G , Brown CH , Howe GW , Sepùlveda M‐J , et al. Re‐envisioning, retooling, and rebuilding prevention science methods to address structural and systemic racism and promote health equity. Prev Sci. 2022:1‐14. 10.1007/s11121-022-01439-4 PMC9554395 36223046

[82] *Braidwood Management, Inc. v. Becerra* (15 May 2023), Civil Action No. 4:20‐cv‐00283‐O. United States Court of Appeals, Fifth Circuit. Northern District of Texas. 2022. Accessed 16 May 2023. Available at: https://caselaw.findlaw.com/us‐dis‐crt‐n‐d‐tex‐for‐wor‐div/1911374.html

[83] Paltiel AD , Ahmed AR , Jin EY , McNamara M , Freedberg KA , Neilan AM , et al. Increased HIV transmissions with reduced insurance coverage for HIV pre‐exposure prophylaxis: potential consequences of Braidwood Management v. Becerra. Open Forum Infect Dis. 2023;10(3):ofad139. 37008565 10.1093/ofid/ofad139 PMC10061554

[84] Jennings Mayo‐Wilson L , Benotsch EG , Grigsby SR , Wagner S , Timbo F , Poteat T , et al. Combined effects of gender affirmation and economic hardship on vulnerability to HIV: a qualitative analysis among U.S. adult transgender women. BMC Public Health. 2020;20(1):782. 32456674 10.1186/s12889-020-08902-3 PMC7249630

[85] Felt D , Xu J , Floresca YB , Fernandez ES , Korpak AK , Phillips G , et al. Instability in housing and medical care access: the inequitable impacts of the COVID‐19 pandemic on U.S. transgender populations. Transgend Health. 2023;8(1):74–83. 36824386 10.1089/trgh.2021.0129 PMC9942178

[86] Kirzinger A , Kearney A , Montero A , Sparks G , Dawson L , Brodie M . KFF/The Washington Post Trans Survey. San Francisco, CA: KFF (Kaiser Family Foundation); 2023.

[87] Fuentes K . Sex worker collectives within the whorearchy: intersectional inquiry with sex workers in Los Angeles, CA. Affilia. 2023;38(2):224–243.

[88] Breslow AS , Wojcik H , Cox R, Jr. , Tran NM , Brewster ME . Toward nonbinary nuance in research and care: mapping differences in gender affirmation and transgender congruence in an online national U.S. survey. Transgend Health. 2021;6(3):156–163. 34159259 10.1089/trgh.2020.0038 PMC8215399

[89] Wesp LM , Malcoe LH , Elliott A , Poteat T . Intersectionality research for transgender health justice: a theory‐driven conceptual framework for structural analysis of transgender health inequities. Transgend Health. 2019;4(1):287–296. 31663035 10.1089/trgh.2019.0039 PMC6818474

[90] Beischel WJ , Gauvin SEM , van Anders SM . “A little shiny gender breakthrough”: community understandings of gender euphoria. Int J Transgend Health. 2022;23(3):274–294. 35799953 10.1080/26895269.2021.1915223 PMC9255216

[91] Wiegand A . Barred from transition: the gatekeeping of gender‐affirming care during the gender clinic era. Intersect. 2021;15(1):1‐11.

[92] Schoenbaum H . Republican states aim to restrict transgender health care in first bills of 2023. Raleigh, NC: Associated Press; 2023.

[93] Gill‐Peterson J . Doctors who? Radical lessons from the history of DIY transition. Baffler. 2022;65:7‐15. Accessed 9 August 2023. Available at: https://thebaffler.com/salvos/doctors‐who‐gill‐peterson.

[94] Andrzejewski J , Dunville R , Johns MM , Michaels S , Reisner SL . Medical gender affirmation and HIV and sexually transmitted disease prevention in transgender youth: results from the survey of today's adolescent relationships and transitions, 2018. LGBT Health. 2021;8(3):181–189. 33566718 10.1089/lgbt.2020.0367 PMC8059356

[95] Coleman E , Radix AE , Bouman WP , Brown GR , de Vries ALC , Deutsch MB , et al. Standards of care for the health of transgender and gender diverse people, version 8. Int J Transgend Health. 2022;23(Suppl 1):S1–S259. 36238954 10.1080/26895269.2022.2100644 PMC9553112

[96] de Vries E , Kathard H , Müller A . Debate: why should gender‐affirming health care be included in health science curricula? BMC Med Educ. 2020;20(1):51. 32059721 10.1186/s12909-020-1963-6 PMC7023748

[97] Dubin SN , Nolan IT , Streed CG, Jr. , Greene RE , Radix AE , Morrison SD . Transgender health care: improving medical students' and residents' training and awareness. Adv Med Educ Pract. 2018;9:377–391. 29849472 10.2147/AMEP.S147183 PMC5967378

[98] Elopre L , Boutwell A , Gordon B , Johnson B , Marrazzo J , Van Der Pol B , et al. PrEP service delivery preferences of black Cis‐gender women living in the Southern United States. AIDS Behav. 2022;26(11):3469–3479. 35445992 10.1007/s10461-022-03691-9 PMC9022049

[99] Dombrowski JC , Golden MR , Barbee LA , Khosropour CM . Patient disengagement from an HIV preexposure prophylaxis program in a sexually transmitted disease clinic. Sex Transm Dis. 2018;45(9):e62–e64. 29485544 10.1097/OLQ.0000000000000823 PMC6086745

[100] Bhatia R , Modali L , Lowther M , Glick N , Bell M , Rowan S , et al. Outcomes of preexposure prophylaxis referrals from public STI clinics and implications for the preexposure prophylaxis continuum. Sex Transm Dis. 2018;45(1):50–55. 28876282 10.1097/OLQ.0000000000000690

[101] Iott BE , Loveluck J , Benton A , Golson L , Kahle E , Lam J , et al. The impact of stigma on HIV testing decisions for gay, bisexual, queer and other men who have sex with men: a qualitative study. BMC Public Health. 2022;22(1):471. 35264132 10.1186/s12889-022-12761-5 PMC8908600

[102] Golub SA . PrEP stigma: implicit and explicit drivers of disparity. Curr HIV/AIDS Rep. 2018;15(2):190–197. 29460223 10.1007/s11904-018-0385-0 PMC5884731

[103] Mayer KH , Agwu A , Malebranche D . Barriers to the wider use of pre‐exposure prophylaxis in the United States: a narrative review. Adv Ther. 2020;37(5):1778–1811. 32232664 10.1007/s12325-020-01295-0 PMC7467490

[104] Edelman EJ , Moore BA , Calabrese SK , Berkenblit G , Cunningham CO , Ogbuagu O , et al. Preferences for implementation of HIV pre‐exposure prophylaxis (PrEP): results from a survey of primary care providers. Prev Med Rep. 2019;17:101012. 31890474 10.1016/j.pmedr.2019.101012 PMC6926349

[105] Blackstock OJ , Moore BA , Berkenblit GV , Calabrese SK , Cunningham CO , Fiellin DA , et al. A cross‐sectional online survey of HIV pre‐exposure prophylaxis adoption among primary care physicians. J Gen Intern Med. 2017;32(1):62–70. 27778215 10.1007/s11606-016-3903-z PMC5215171

[106] Sewell WC , Powell VE , Ball‐Burack M , Mayer KH , Ochoa A , Marcus JL , et al. Brief Report: “I didn't really have a primary care provider until I got PrEP”: patients' perspectives on HIV preexposure prophylaxis as a gateway to health care. J Acquir Immune Defic Syndr. 2021;88(1):31–35. 34397743 10.1097/QAI.0000000000002719 PMC8369038

[107] Calabrese SK , Krakower DS , Mayer KH . Integrating HIV preexposure prophylaxis (PrEP) into routine preventive health care to avoid exacerbating disparities. Am J Public Health. 2017;107(12):1883–1889. 29048955 10.2105/AJPH.2017.304061 PMC5678380

[108] Marcus JL , Levine K , Grasso C , Krakower DS , Powell V , Bernstein KT , et al. HIV preexposure prophylaxis as a gateway to primary care. Am J Public Health. 2018;108(10):1418–1420. 30024802 10.2105/AJPH.2018.304561 PMC6137783

[109] Faro EZ , Mantell JE , Gonzalez‐Argoti T , Hoffman S , Edelstein Z , Tsoi B , et al. Implementing PrEP services in diverse health care settings. J Acquir Immune Defic Syndr. 2022;90(S1):S114–S128. 35703763 10.1097/QAI.0000000000002971 PMC9204802

[110] Hill SV , Westfall AO , Coyne‐Beasley T , Simpson T , Elopre L . Identifying missed opportunities for human immunodeficiency virus pre‐exposure prophylaxis during preventive care and reproductive visits in adolescents in the Deep South. Sex Transm Dis. 2020;47(2):88–95. 31934955 10.1097/OLQ.0000000000001104

[111] Kelly JA . Ten things we need to do to achieve the goals of the end the HIV epidemic plan for America. J Acquir Immune Defic Syndr. 2019;82(Suppl 2(2)):S94–S98. 31658194 10.1097/QAI.0000000000002166 PMC6822383

[112] Jenkins WD , Phillips G, 2nd , Rodriguez CA , White M , Agosto S , Luckey GS . Behaviors associated with HIV transmission risk among rural sexual and gender minority and majority residents. AIDS Care. 2023;35(10):1452‐1464. 36803272 10.1080/09540121.2023.2179592

[113] Harrison SE , Paton M , Muessig KE , Vecchio AC , Hanson LA , Hightow‐Weidman LB . “Do I want PrEP or do I want a roof?”: social determinants of health and HIV prevention in the Southern United States. AIDS Care. 2022;34(11):1435‐1442. 35109734 10.1080/09540121.2022.2029816 PMC9343473

[114] Connell CL , Wang SC , Crook L , Yadrick K . Barriers to healthcare seeking and provision among African American adults in the rural Mississippi Delta region: community and provider perspectives. J Community Health. 2019;44(4):636–645. 30661152 10.1007/s10900-019-00620-1 PMC6612316

[115] Wilson EC , Arayasirikul S , Johnson K . Access to HIV care and support services for African American transwomen living with HIV. Int J Transgend. 2013;14(4):182–195. 24817835 10.1080/15532739.2014.890090 PMC4012687

[116] Reback CJ , Ferlito D , Kisler KA , Fletcher JB . Recruiting, linking, and retaining high‐risk transgender women into HIV prevention and care services: an overview of barriers, strategies, and lessons learned. Int J Transgend. 2015;16(4):209–221. 27110227 10.1080/15532739.2015.1081085 PMC4838285

