# Supplementary material for: HIV pre‐exposure prophylaxis programme preferences among sexually active HIV‐negative transgender and gender diverse adults in the United States: a conjoint analysis
Source: J Int AIDS Soc. 2024 Feb 8;27(2):e26211. doi: 10.1002/jia2.26211 (PMC10853582; doi:10.1002/jia2.26211)
Supplement: Supplementary file 1 — Supporting Information [file JIA2-27-e26211-s001.docx]

**Supplemental Table 1. The International Society for Pharmacoeconomics and Outcomes Research (ISPOR) Good Research Practices for Conjoint Analysis Task Force Checklist for Conjoint Analysis in Health Care.**

| **Checklist Step** | **How step was addressed** |
| --- | --- |
| 1. Research question | - This study measured participants’ preferences for hypothetical HIV pre-exposure prophylaxis (PrEP) program attributes and preferred program overall. - The perspective of PrEP-naïve and PrEP-experienced transgender and gender-diverse adults in the United States was examined. - A conjoint analysis was appropriate to measure respondents’ willingness to trade off attributes of multi-attribute PrEP service delivery models. |
| 1. Attributes and levels | - Attributes selected based on literature review of factors shown to influence PrEP delivery among priority populations in the United States and expert consultation regarding next-generation PrEP modalities in development as of March 2021. Priority populations included transgender persons, cisgender sexual minority men, Black cisgender women, and people who use drugs. - In accordance with good research practice, the maximum number of levels per attribute was limited to more than three. - Attribute levels were reviewed for appropriateness and wording by research team members with expertise in PrEP service delivery (two board-certified infectious disease physicians) and members of the study’s community advisory board. |
| 1. Construction of tasks | - An example conjoint task was provided. - Fractional factorial design of the attributes and levels was used (i.e., respondents were shown a fraction of the full factorial design) to maximize the number of data points and the coverage across potential PrEP programs, while minimizing cognitive burden for the respondents. - Each task consisted of five attributes per profile and two profiles per task. - Respondents were asked to choose the more preferred profile between each pair presented. - An opt-out option was included in each task. - No exclusions or prohibited pairs were included. |
| 1. Experimental design | - Proprietary Qualtrics® conjoint analysis program was used a randomized balance design approach similar to Sawtooth’s Balanced Overlap Design. - Design was near-orthogonal (levels varied independently) and near-balanced (each level appeared equally across the conjoint analysis experiment):   - Step 1: Algorithm randomly generated profiles for each task.   - Step 2: Algorithm checked each version of the experiment for balance across each level.     - Complete balance was not forced; however, the algorithm confirmed the difference between the level shown the most and the level shown the least was less than two deviations     - Versions that did not meet these conditions were refactored until they complied with balance rules.   - Step 3: The algorithm continued until the desired number of versions was generated. - Each participant completed five choice tasks. |
| 1. Preference elicitation | - Paired comparisons method   - Shown to have highest reliability relative to the full profile method and the trade-off method (Reibstein D, et al. 1988; Rao VR, 2014). - A brief description of PrEP followed by five tasks and instructions to select the preferred program profile for each task. - Tasks were presented in random order to minimize effect bias. |
| 1. Instrument design | - Full online survey included the conjoint analysis experiment and measures related to sociodemographic characteristics; gender affirmation; sexual behaviors; knowledge, attitudes, and experiences related to PrEP and other health care. - Full survey was pilot tested by community advisory board to assess the level of cognitive burden and time to completion. |
| 1. Data collection | - Calculated a sample size of 300 respondents for aggregate-level estimation of main effects (Orme BK, 2020). - Transgender adults in the United States who self-reported negative or unknown HIV status and recent sexual activity were recruited April—June 2022 via social media, LGBTQ+ and transgender health listservs, word of mouth, and email invitations. - Study procedures were approved by the University of Pennsylvania Institutional Review Board. - National online self-administered survey via Qualtrics® online platform. |
| 1. Statistical analysis | - Descriptive statistics were computed for the study population and comparisons were made across participant characteristics by respondents’ self-reported PrEP status (PrEP-experienced vs PrEP-naïve). - Hierarchical Bayes estimation and multinomial logistic regression were used to measure respondents’ part-worth utility scores (regression coefficients) and characterize the attribute importance, preferences, and optimal program attributes for the study population and by respondents’ self-reported PrEP status (PrEP-experienced vs PrEP-naïve). - Statistical analyses not performed in Qualtrics were performed using Stata version 15 (StataCorp LLC, 2017). |
| 1. Results and conclusions | - Results indicated that out-of-pocket cost had the highest attribute importance score followed by the ability to bundle with gender-affirming hormone therapy services. PrEP-experienced respondents preferred PrEP program service delivery in primary care settings; however, PrEP-naïve respondents preferred pharmacies. - 95% confidence intervals indicated the degree of uncertainty and precision for a given statistical estimate. - Results were compared to published studies. - Limitations included potential variability in respondents’ comprehension for each attribute while ranking program scenarios; sampling bias due social media recruitment and high rates of PrEP awareness, bias due to task ordering. |
| 1. Study presentation | - This study demonstrated good research practices. - The unique contributions of the study were discussed. |

**Notes**: Checklist steps are based on the consensus-based recommendations for good research practices published by the International Society for Pharmacoeconomics and Outcomes Research Conjoint Analysis Task Force (Bridges et al., 2011; Johnson et al., 2013) and adapted from the general framework for good research practices for conducting a discrete-choice experiment published by Janssen et al., 2018.
